# *BAALC* and *ERG* Expression in Egyptian Patients with Acute Myeloid Leukemia, Relation to Survival and Response to Treatment

**DOI:** 10.3889/oamjms.2016.058

**Published:** 2016-05-22

**Authors:** Aml Soliman, Asmaa Abdel Aal, Reham Afify, Noha Ibrahim

**Affiliations:** *Cairo University, Clinical Pathology, Cairo, Egypt*

**Keywords:** *BAALC*, *ERG*, gene expression, Quantitative real-time RT-PCR, AML

## Abstract

**AIM:**

Aim was to detect Brain and Acute Leukemia, Cytoplasmic (*BAALC*) and ETS-related gene (*ERG*) expression in patients with acute myeloid leukemia (AML) as well as to study their biologic and prognostic impact on the disease outcome and survival.

**PATIENTS AND METHODS:**

The current study was carried out on 44 patients with denovo acute myeloid leukemia, as well as 44 age and sex matched controls. The quantitative real-time reverse transcription-polymerase chain reaction (RT-PCR) assay was performed for estimation of *BAALC* and *ERG* expression.

**RESULTS:**

The current study was carried out on 44 patients with denovo acute myeloid leukemia, as well as 44 age and sex matched controls. The quantitative real-time reverse transcription-polymerase chain reaction (RT-PCR) assay was performed for estimation of *BAALC* and *ERG* expression. *BAALC* was expressed in 36 (81.82%) of AML cases versus 10 (22.72%) of the control group which was highly statistically significant (P < 0.001). While *ERG* was positive in 39(88.64%) of cases and 8(18.18 %) of controls and that was also highly statistically significant (P < 0.001).

**CONCLUSION:**

Further researches still needed to clarify the role of *BAALC* and *ERG* in the pathogenesis of leukemia and their importance as targets for treatment of AML.

## Introduction

Acute myeloid leukemia (AML) is a heterogeneous disease with respect to clinical picture and therapeutic outcome, partly reflected by differences in molecular and cytogenetics [1-3].

Clonal cytogenetic abnormalities are one of the most important factors predicting clinical outcome in acute myeloid leukemia and are used to guide risk-adapted treatment strategies [4-6]. Deregulated expression of genes coding transcription factors involved in cell proliferation, survival or differentiation is known to be implicated in the process of leukemogenesis [7-9]. Brain and acute leukemia, the cytoplasmic gene (*BAALC*) and ETS-related gene (*ERG*) are examples of these transcription factors [10-12].

The brain and acute leukemia, cytoplasmic (*BAALC*), which maps on chromosome 8 at 8q22.3, was originally observed in the neuroderm and its expression was reported as a hematopoietic precursor, such as the early hematopoietic cells of a cluster of differentiation 34+ [8]. *BAALC* expression was higher in bone marrow compared to blood. In addition, high levels of *BAALC* expression were present in leukemic blasts in subsets of acute lymphoblastic leukemia (ALL) and AML patients with a normal karyotype [13], it may act as an adverse prognostic factor through prompting proliferation and inhibiting apoptosis in leukemia cells [14].

*BAALC* gene is considered as a marker of early hematopoietic progenitor cells. High levels of *BAALC* expression were found in AML patients with trisomy 8, as well as in a subset of cytogenetically normal – AML(CN-AML) patients in which it was considered as a poor prognostic factor [15, 16]. The role of *BAALC* in leukemogenesis is not fully understood, but it was proposed that *BAALC* blocks myeloid differentiation [15].

*ERG* gene, located on chromosome band 21q22, belongs to the ETS family of transcription factors required for normal hematopoiesis. *ERG* plays an important role in early hematopoiesis and hematopoietic stem cell (HSC) maintenance [17].

It is a transforming proto-oncogene that is expressed in stem cells and endothelial cells. It is frequently overexpressed in AML patients with complex karyotypes and amplification of chromosome 21 [18].

The *ERG* gene has been found to be involved in atypical chromosomal rearrangements with alterations of the transcription factor in several cancers. Aberrant expression of full-length *ERG* protein has been found in acute myeloid leukemia and acute T-lymphoblastic leukemia [19, 20]. Chromosomal rearrangements driving the formation of *EWS/ERG* and *FUS/ERG* fusion proteins have been described in a subset of Ewing sarcoma [21, 22] and in acute myeloid leukemia [23, 24]. In acute leukemia, high *ERG* mRNA expression levels are an independent prognostic factor. Studies have demonstrated that increased *ERG* expression is associated with poor prognosis in cytogenetically normal AML [25] and in adult T-ALL [10].

Most of the previous researches were concerned about levels and prognostic effect of *BAALC* and *ERG* in CN-AML patients while data about their incidence in AML and relation to cases with abnormal karyotype were lacking.

We sought to determine the prognostic impact of the expression of *BAALC* and *ERG* in de novo AML patients.

The objective of this study was to detect the incidence of *BAALC* and *ERG* in a group of denovo AML patients with abnormal karyotype in comparison to normal individuals, their levels and distribution among AML FAB subtypes as well as to study their impact on the disease outcome and reliability in detection of minimal residual disease in relation to the cytogenetic markers.

## Patients and Methods

The current study was carried out on 44 patients with denovo acute myeloid leukemia (AML), in the period between, July 2011 and July 2012 among cases referred to nuclear medicine and oncology unit of Kasr Al Ainy School of medicine, Cairo University with follow-up period of 18 months, as well as 44 age and sex matched controls. They were patients suspected to have hypersplenism or idiopathic thrombocytopenia coming for BM aspirate. Cases were diagnosed according to WHO criteria [25]. The diagnosis of AML was based on morphological and phenotypic data. Subtypes according to the French– American–British classification were available for all the patients.

Patients were 21 males and 23 females. Age ranged from 21 to 65 years. The control group included 17 male and 27 females with no medical history of any type of cancer. Their ages ranged between 22 and 50 years. All patients and controls were analyzed for clinical and laboratory findings, including full history taking, clinical examination, routine laboratory investigations, LDH, abdominal ultrasound for detection of organomegaly and lymphadenopathy. The patients were subjected as well to cytochemical, immunophenotypic and cytogenetic analysis to confirm the diagnosis and to divide the patients into their subtypes. Local institutional research board approval as well as written informed consent was obtained from all the participants before including them in the study.

All patients included in the study were treated according to the protocol of the nuclear medicine and oncology department, Cairo University, using ongoing induction and consolidation regimens for treatment of adult AML cases.

*Induction of remission:* Patients were subjected to 7-3 protocol [26] for induction of remission: Novantrone: 12 mg/m^2^, IV on day 1and 3. ARA – C: 100 mg/m^2^, continuous IV infusion, from day l-7. If remission is not achieved, this protocol was repeated again. If no or minimal response, patients were shifted to high dose chemotherapy. Induction therapy for acute promyelocytic leukemia (PML) included oral administration of all-trans-retinoic acid (ATRA) 45 mg/m^2^/day until induces remission.

*Consolidation [27]:* High-dose ARA-C for 4 cycles. ARA-C: 2g/m^2^, over 2 hour’s infusions, every 12 hours on days 1-4. Significant association to relapse-free survival and overall survival were estimated for studied genes at a median follow-up of 18 months.

Complete remission (CR) was defined as recovery of morphologically normal BM and peripheral blood cells with white cell counts ≥ 1,500/L, and platelets ≥ 100,000/L, less than 5% BM blasts and no evidence of extramedullary leukemia. Relapse was defined by ≥ 5% BM or peripheral blood blasts, or development of extramedullary leukemia in patients with previously documented CR. Relapse-free survival (RFS) was measured from the date of CR until the date of relapse or death [28].

### Collection and Processing Of Samples

Gene expression was analyzed using quantitative real-time RT-PCR. Three ml of peripheral blood (of AML cases) and bone marrow samples (of control group) were collected on sterile ethelenediaminetetraacetic acid (EDTA) vacutainers. Mononuclear cells were enriched by Ficoll-Hypaque gradient and total RNA was extracted from freshly separated cells using Qiagen RNeasy mini spin column (RNeasy mini kit, Qiagen, Valencia, CA, USA) according to the manufacturer instructions.

Quantitative real-time RT-PCR assay was performed for estimation of *BAALC* and *ERG* expression. *BAALC* and *ERG* mRNA expression levels were estimated from the corresponding gene-specific calibration curves. The level *GAPDH* expression was measured for each sample using *GAPDH* standard curve.

### DNA Synthesis and Real-Time PCR Quantitation of BAALC and ERG mRNA Expression

One microgram of the total RNA was transcribed into cDNA using random hexamers (High-capacity cDNA kit; Applied Biosystems). An aliquot of the cDNA was used for quantitative PCR amplification by Gene Amp 7500 Sequence Detection System (Applied Biosystems), using 50 μl reaction mix containing 5μl cDNA, 25 μl TaqMan Universal PCR master mix, 2.5μl primer-probe mix using the following primers and probes:

BAALC (F): 5’-GCCCTCTGACCCAGAAACAG-3’, BAALC (R): 5’-’CTTTTGCAGGCATTCTCTTAGCA-3’, BAALC Probe: FAM-5’-CTCTTTTAGCCTCTGTGGTCTGAAGGCCAT-3’-TMRA.
ERG (F): 5’-AACGAGCGCAGAGTTATCGT-3’, ERG (R): 5’-GTGAGCCTCTGGAAGTCGTC-3’, ERG probe: FAM-5’-GGAGTACAGACCATGTGCGGCAGTG-3’-TMRA
GAPDH (F): 5’-GAAGGTGAAGGTCGGAGTC-3’, GAPDH (R): 5’-GAAGATGGTGATGGGATTTC -3, GAPDH probe: FAM-5
-CAAGCTTCCCGTTCTCAGCC-3’-TMRA and 17.5 μl H_2_O.

The thermal cycling conditions included: 10 min at 95 ºC followed by 45 cycles of denaturation for 15 min at 95ºC, annealing/extension at 60ºC for 1 min. A negative control (5 μl water instead of cDNA) was included in every TaqMan plate. The results were presented as the relative quantification levels of *BAALC* and *ERG* in relation to the internal control *GAPDH* using the comparative cycle threshold (CT) method (2^-ΔΔ^CT). The cycle number difference e.g (ΔC_T_ = *GAPDH* − *BAALC*), was estimated for each case then 2^-ΔΔ^CT was calculated.

The threshold cycle data (Ct) and baselines were determined using auto settings. The relative quantification of *BAALC* and *ERG* expression was calculated using the comparative CT method (2–ΔΔCT) where ΔΔCT is the difference of ΔCT value between leukemia and the control (ΔΔCT = ΔCT leukemia gene – ΔCT control gene), and ΔCT is the difference of CT value between the target (gene) and endogenous reference (GAPDH) gene (ΔCT = CT Target gene – GAPDH gene).

### Statistics

A pre-designed SPSS (Statistical Package for Social Science Version 17) file was used for data entry and analysis. The following tests were used: both unpaired and paired t - test, with 95 % confidence intervals (95% CI), comparison of nonparametric quantitative data in two different groups using their mean rank performed by Mann–Whitney (Z). Chi-square was used for comparison of qualitative variables. Patients with expression values greater than the median of all samples were classified as high *BAALC* expression. *ERG* overexpression was defined as transcript level greater than the 75^th^ percentile of all measurements. Correlation between various variables was done using Pearson moment correlation equation for linear relation. P value less than 0.05 was considered statistically significant and less than 0.01 was considered highly statistically significant.

No pre-study power calculation was performed. However, based on the number of subjects and controls included in this study, the sample size provides 95.5 % power to detect a minimal difference of 9.0% with an alpha level of 0.05.”

## Results

The present study was carried out on 44 patients with denovo AML, as well as 44 age and sex matched controls. The clinical and demographic data of the studied groups revealed no statistically significant differences between the 2 studied groups. The results of immunophenotyping and cytogenetics were obtained from the main laboratory of Kasr Alainy hospital. We selected the cases of M2 with t (8; 21), M3 with t (15; 17), and M4 with inversion 16, and M5 with t (9; 11). While cytogenetic results of M1 cases were not available. Twelve patients (27.2%) achieved complete remission following consolidation chemotherapy while 32 patients (72.8%) did not. The overall survival of the patients ranged between 2 and 18 months. Relapse-free survival ranged between 0 and 18 months.

*BAALC* was expressed in 36 (81.82%) of AML cases versus 10 (22.72%) of the control group which was highly statistically significant (P < 0.001). While *ERG* was positive in 39 (88.64%) of cases and 8 (18.18%) of controls and that was also highly statistically significant (P < 0.001). The distribution of the positive cases among FAB subtypes were as follows:

For *BAALC* gene positive cases, 14/17 patients (82%) were M1, 5/7 patients (71%) were M2, 9/10 patients (90%) were M3, 4/5 patients (80%) were M4, 4/5 patients (80%) were M5.

For *ERG* gene positive cases, 16/17 patients (94%) were M1, 5/7 patients (71%) were M2, 10/10 patients (100%) were M3, 4/5 patients (80%) were M4, 4/5 patients (80%) were M5.

The real-time results of the studied genes expressed as CT were summarized in Table 1.

**Table 1:** Results of ΔCT of *BAALC* and *ERG* genes in patients and controls

	Patients (44)		Controls (44)		P value

	Range	Mean ± SD	Range	Mean±SD	
*BAALC*	26 - 45.5	35 ± 3.8	37.8 - 49.5	42.1 ± 8.2	0.045 (S)

*ERG*	32.7- 46.6	39.7 ± 2.6	39.8 - 42.6	41.8 ± 1.1	0.064 (NS)

GADPH	26.9 - 46	32.6 ± 1.6	29.2 - 40.1	33.3 ± 3.4	0.65 (NS)

The lower values of CT in AML cases compared to CT values in controls mean higher levels of *BAALC* and *ERG* genes in the cases as the numerical value of the CT is inversely related to the amount of amplicon in the reaction.

Follow-up of the patients revealed 12 cases of CR and 32 with the unfavorable outcome; 17 showed partial recovery (PR), 8 cases relapsed and 7 patients died.

Correlations between *BAALC* and *ERG* genes expression and other prognostic factors and treatment outcome were summarized in Table 2. Highly significant correlation was detected between positive genes expression and the presence of bulky tumor and organomegaly.

**Table 2:** Correlation between the results of *BAALC* and *ERG* genes expression and prognostic factors in patients

	*BAALC*		*ERG*	

	r	p	r	p
Age	0.06	0.07 [NS]	0.06	0.07 [NS]
Sex	0.06	0.41 [NS]	0.08	0.34 [NS]
Organomegaly	0.56	P < 0.001 [HS]	0.55	P < 0.001 [HS]
Presence of bulky tumor	0.73	P < 0.001 [HS]	0.63	P < 0.001 [HS]
High LDH level	0.03	0.066 [NS]	0.03	0.08 [NS]

The levels of each gene were expressed as 2^-ΔΔ^CTi.e.number of fold increased above the mean level of the control group. Patients were divided into two groups according to these levels (high and low) as described previously in a statistic paragraph. *BAALC* levels median and range were 1479.8 (220.8-7550.2) for high levels group and 10.1 (0.13-54.9) for low levels group. While levels of *ERG* were 425.5 (27-1985) and 2.44 (0.021-8.17) for high and low levels groups respectively. Association of *BAALC* and *ERG* expression levels with clinical characteristics were summarized in Table 3.

**Table 3:** Association of *BAALC* and *ERG* expression levels with clinical characteristics

	*BAALC* expression (N = 36 patients)			*ERG* expression (N = 39patients)		
	High	Low	P value	High	Low	P value
-No	24	12		24	15	
-%	66.7	33.3		61.5	38.5	
						
-Age (years)						
Median	42	44		43	48	
Range	20-62	21-65	0.65 (NS)	20-58	21-65	0.76 (NS)
						
-FAB classification						
M1	9	5		3	2	
M2	3	2		9	7	
M3	6	3		6	4	
M4	3	1		3	1	
M5	3	1	0.54 (NS)	3	1	0.59 (NS)
						
-Leucocyte count						
Median	21.9	13.6		22.4	12.1	
Range	3.0 -32	3.2-24	0.03 (S)	3.2 -32	3-20.5	0.02 (S)
						
-Hg level						
Median	7.5	8		7.8	8.9	
Range	3.1-8.5	5.2-10	0.64 (NS)	3.0-7.4	6.1-10.0	0.75 (NS)
						
-Platelet count						
Median	100	120		90	110	
Range	30-110	36-130	0.73 (NS)	32-100	36-135	0.56 (NS)
						
-BM blasts%						
Median	85	80	0.67(NS)	83	77	
Range	22-98	21-90		21-96	20-89	0.43 (NS)
-Response to treatment	2	10	0.03(S)	3	9	0.04 (S)
-Relapse-free survival Median(months)	3	16	P < 0.001 [HS]	3	16	P < 0.001 [HS]
-Overall survival Median (months)	6	18	P < 0.001 [HS]	6	18	P < 0.001 [HS]

There were statistically no significant differences between the 2 studied groups regarding the *BAALC* gene expression before and after treatment (P value=0.86) or the *ERG* gene expression before and after treatment (P value=0.75) (Table 4).

**Table 4:** Results of *BAALC* and *ERG* expression in patients before and after treatment

	*BAALC*		*ERG*	

	high	low	high	low
Median	1479.8	10.2	425.5	2.4
Range	220.7-7550.2	0.13-54.9	27.3-1985.9	0.02-8.2

The multivariate regression test had revealed that *BAALC* and *ERG* are independent risk factors for acute leukemia, because (Table 5). Although we abolished the effect of bad prognostic factors (age > 60 years, high serum LDH and WBC levels, high bone marrow blast percentage, presence of a bulky tumor), still high levels of these genes are associated with lower CR, RFS, and shorter OS.

**Table 5:** Multivariate regression analysis for *BAALC* and *ERG* expression with the prognostic markers

	Patients groups	OR	HR	95% CI	P value
-Complete remission	*BAALC* expression, high versus low	4.3	-	3.5-9.7	0.03 (S)
	*ERG* expression, high versus low	3.9	-	3.1-8.5	0.04 (S)
-RFS					
	*BAALC* expression, high versus low		2.1	1.5-2.8	0.02 (S)
	*ERG* expression, high versus low		1.5	1.1-2.7	0.02 (S)
-OS					
	*BAALC* expression, high versus low		2.2	1.7-3.5	0.03 (S)
	*ERG* expression, high versus low		2.8	2.4-4.5	0.04 (S)

## Discussion

*BAALC* and *ERG* are transcription factors involved in the process of hematopoiesis subsequently their deranged expression is associated with leukemogenesis and its prognosis. We estimated their levels in BM of normal individuals and peripheral blood of leukemic cases as their expression is restricted to the progenitor cells in the BM and down-regulated with cellular maturation. The present study revealed 36/44 (81.82%) *BAALC* positive AML cases compared to10/44 (22.72%) of the control group (P < 0.001). While *ERG* was positive in 39 (88.64%) of cases and 8 (18.18 %) of controls and that was also highly significant (P < 0.001). Positive cases were associated with bulky tumor and organomegaly. That was higher than the incidence of *BAALC* detected by Balatzenko et al [29]; 53/91 (58.2%).

High levels of *BAALC* and *ERG* were detected almost concomitantly in 54.5% of cases. They showed significant positive correlation to white cell counts. Marcucci et al. [19], also detected concomitant high *BAALC* and *ERG* levels in 25% of AML-NC cases with positive correlation to blast counts.

Eid et al., [25], detected high expression of *BAALC* in 70% of patients and its expression did not correlate with the clinical parameters of patients. *ERG* was high in 33.3% of patients and its expression was associated with lower ages and higher white cell counts.

The distribution of positive cases and cases with high *BAALC* and *ERG* among FAB subtypes did not show any significant difference. Subsequently, no association with their cytogenetic abnormalities was found. That was also concomitant with results of Eid et al, [25] but different from that of Baldus et al., 2006[10] who found a significant association between *BAALC* and M0/M1.

Heuser et al. [30] have shown that *BAALC* expression hinders cell differentiation, but does not promote cell proliferation. They believed that for the onset of leukemia another factor must exist to promote uncontrolled proliferation.

As regard treatment outcome high levels of *BAALC* and *ERG* were associated with lower incidence of CR (P = 0.03 and 0.04 respectively) and shorter RFS and OS (P<0.001).

Multivariable analysis demonstrated that *BAALC* and *ERG* genes over-expression are an independent predictor of low rate of CR, shorter RFS, and OS. These results are in consistent with Metzeler et al., 2009 [12], who found that high transcript levels of *ERG* and *BAALC* were predictors for inferior OS and a lower rate of CRs. They also described significant positive correlations between the expression levels of the two genes and a third one (*MN1*). In agreement with these results, Baldus et al. 2003 [8], 2006 [10] demonstrated a significant association between high *BAALC* expression and resistance of AML to therapy and low CR. Moreover, they found high *BAALC* to be an independent predictor of relapse. Marcucci et al., 2005 [19] also demonstrated that increased *ERG* expression was associated with poor prognosis in CN-AML.

Schwind et al., 2010 [31], found that expression levels of both *BAALC* and *ERG* were the only factors significantly associated with overall survival upon multivariable analysis.

In order to investigate the applicability of *BAALC* and *ERG* in the detection of MRD, we measured their levels again in patients achieved CR for the first time. Although their levels decreased in each examined case but their reduction did not reach statistical significant difference while the cases showed normal morphological and cytogenetic examinations.

Weber et al., [32] correlated *BAALC* expression levels of the diagnostic samples with those of the first sample showing complete molecular remission (CMR) defined by NPM1 mutational status. They found, no significant difference of *BAALC* expression levels in those nine patients during treatment. However, 13 patients with *BAALC* overexpression at diagnosis showed a strong reduction in its mean expression levels at first CMR. Moreover, in four cases a molecular relapse was detected based on elevated *BAALC* expression levels within 37–149 days before morphological relapse. Conflicting results had appeared when they correlated *BAALC* expression levels with other mutated genes used in MRD detection.

Due to limited financial support, we could not estimate the changes in *BAALC* and *ERG* levels during the course of treatment and follow-up. Their levels might decrease gradually after CR. To overcome the previous conflicting results, larger scale researches must be done on AML cases to determine applicability and sensitivity of *BAALC* in the detection of MRD.

As regard *ERG*, the levels in AML cases did not differ significantly from theirs in normal BM. So the determination of cut-off value to detect MRD will not be applicable.

Further researches still needed to clarify the role of *BAALC* and *ERG* in the pathogenesis of leukemia and their importance as targets for treatment of AML that could be promising due to their high incidence of expression in AML.
